# Prevalence and antibiotic resistance of bacteria isolated from the cerebrospinal fluid of neurosurgical patients at Peking Union Medical College Hospital

**DOI:** 10.1186/s13756-018-0323-3

**Published:** 2018-03-20

**Authors:** Jian-bo Chang, Hao Wu, He Wang, Bai-tao Ma, Ren-zhi Wang, Jun-ji Wei

**Affiliations:** 1 Department of Neurosurgery, Peking Union Medical College Hospital, Peking Union Medical College, Chinese Academy of Medical Sciences, No.1 Shuaifuyuan, Dongcheng District, Beijing, 100730 China; 2 Department of Laboratory Medicine, Peking Union Medical College Hospital, Peking Union Medical College, Chinese Academy of Medical Sciences, No.1 Shuaifuyuan, Dongcheng District, Beijing, 100730 China

**Keywords:** Prevalence, Antibiotic resistance, Central nervous system infections, Neurosurgery

## Abstract

**Background:**

Postoperative central nervous system infections (PCNSIs) represent a serious complication, and the timely use of antibiotics guided by the identification of the causative pathogens and their antibiotic sensitivities is essential for treatment. However, there are little data regarding the prevalence of PCNSI pathogens in China. The aim of this study is to investigate the features of pathogens in patients with PCNSIs, which could help clinicians to choose the appropriate empirical antibiotic therapy.

**Methods:**

We retrospectively examined the positive CSF cultures in patients who underwent craniotomy between January 2010 and December 2015. We collected data, including demographic characteristics, type of neurosurgery, laboratory data, causative organisms and antimicrobial susceptibility testing results.

**Results:**

A total of 62 patients with 90 isolates out of 818 patients with 2433 CSF culture samples were available for data analysis. The estimated incidence and culture-positive rate of PCNSIs were approximately 0.9 and 7.5%, respectively. The predominant organism was coagulase-negative staphylococci, of which most were methicillin-resistant coagulase-negative staphylococci (MRCoNS). All were susceptible to vancomycin, linezolid, rifampicin and amoxicillin-clavulanate. *Acinetobacter baumannii* was the most frequent causative Gram-negative agent and was resistant to 12 out of 18 antimicrobials tested. The sensitivity rates for tigecycline and minocycline were only 40 and 33%, respectively.

**Conclusion:**

PCNSIs could lead to high mortality. Although the MRCoNS were the predominant organism, the management of *Acinetobacter baumannii* was a major clinical challenge with few effective antimicrobials in PCNSIs.

**Electronic supplementary material:**

The online version of this article (10.1186/s13756-018-0323-3) contains supplementary material, which is available to authorized users.

## Background

Postoperative central nervous system infections (PCNSIs) are relatively rare but serious complication following neurosurgery, especially craniotomies, resulting in poor patient outcomes and increasing the total cost of illness. Craniotomies are used for the treatment of head injury, brain tumor or intracranial hemorrhage, external ventricular device placement, and entriculostomy [1]. The incidence of infections after cranial surgery, mostly meningitis, varies from 0.3 to 7.8% [2, 3]. The diagnosis of PCNSIs requires a high degree of clinical suspicion and lumbar puncture for cerebrospinal fluid (CSF) culture. Once diagnosed, selection of the empirical antibiotic should be guided by knowledge of the causative pathogens and their sensitivities.

The epidemiology of bacterial infections varies with time, geographical distribution, age, race, underlying medical and/or neurosurgical conditions, means of contraction, status of vaccination, and use of antibiotics in the community [4]. Although the primary pathogens in PCNSI are still Gram-positive bacteria, such as *Staphylococcus aureus*, there is a possible trend towards greater numbers of Gram-negative bacteria, especially *Acinetobacter* [5, 6]. Another obvious change is the increased rates of multi-antibiotic resistant strains such as methicillin-resistant *S. aureus* (MRSA) and strains that produce extended-spectrum β-lactamases (ESBL) in recent years [7]. The potential underlying cause is the widespread use of empirical vancomycin and antipseudomonal β-lactam antibiotics [8].

Both the species and the antibiotic resistance profile of bacteria, as well as the blood-brain barrier, are crucial components to consider when selecting the appropriate antibiotics. There have been many recent studies reporting the risk factors for PCNSIs [9–12] in China. Unfortunately, little data regarding prevalence and antibiotic resistance of bacteria have been published. Peking Union Medical College Hospital (PUMCH), founded by the Rockefeller Foundation in 1921, is a university general hospital and tertiary referral center in northern China, where the department of neurosurgery conducts approximately 1200 operations each year. As one of the largest pituitary centers in China, about half operations were endonasal trans-sphenoidal approach [13]. Recently, the average length of stay has been approximately 6 days. This research will report the features of pathogens and investigate the clinical characteristics of post-craniotomy PCNSIs in a single institution over the past 6 years.

## Methods

This study was approved by the Ethics Committee of Peking Union Medical College Hospital (PUMCH). The results of CSF cultures isolated from inpatients were reviewed via a computerized log containing records from January 2010 to December 2015. The data of patients with positive culture isolates were collected from medical records using previously designed standardized evaluation forms (the details showed in Additional file 1) that included demographic characteristics, types of neurosurgery, laboratory data, causative organisms and antimicrobial susceptibility testing. The positive CSF culture result was the main inclusion criteria. The isolation of the same species from the same patient within 7 days was regarded as the same isolate and not counted multiple times. All the patients met clinical diagnostic criteria of nervous central infection [14]. Culture contamination was determined by specialists from the clinical laboratory and neurosurgery, which was based on the identity of the microorganism itself and its clinical features [14–16]. Previous cranial operation, including both elective and emergency procedures, was another inclusion criteria. Patients who underwent a spinal operation or simple wound revision without drilled holes were excluded from the study. Patients with evidence of concomitant chronic meningitis or encephalitis not due to microbial infection were also excluded.

All patients received 1.5 g of a second-generation cephalosporin as prophylactic therapy 1 h before incision and followed by a 24-h postoperative course. Strict precautions were taken throughout the operation, including the use of antibacterial film, to ensure that there was no perioperative breach in sterility [17]. We analyzed the results of antibiotic susceptibility based on the Clinical and Laboratory Standards Institution (CLSI) methods [15]. Pathogenic organisms were considered resistant if they were reported as intermediate or resistant to at least one of the agents, which included cephalosporins, fluoroquinolones, aminoglycosides, carbapenems, extended-spectrum penicillins, macrolides, and β-lactam/β-lactamase inhibitors.

## Results

From 2010 to 2015, approximately 6500 in-hospital patients were treated with approximately 7500 operations in the department of neurosurgery. We obtained 2433 CSF culture samples from 818 patients. Of these, 78 patients with 108 positive isolates were identified, and their medical records were reviewed. Following review, 12 isolates were excluded due to contamination, and four patients were removed because they did not undergo surgery. Finally, 62 patients with 90 isolates met the inclusion criteria. Of them, two patients were failed to obtain the whole medical records and the sensitivity results of five isolates were not available. The incidence of PCNSIs was approximately 0.9% (62/6500) and the estimated culture-positive rate of infection was approximately 7.5% (62/818) and 16 patients with 44 isolates were infected with polymicrobial, in which six patients with more than two species.

Table 1 shows the characteristics of 60 patients with positive CSF cultures. The median days of hospitalization and the infection period postoperation were 46.5 (IQR = 38) days and 9 (IQR = 9) days, respectively. Half of the patients underwent surgery using the endonasal trans-sphenoidal approach, and most were diagnosed with a pituitary adenoma. Most patients treated with craniotomy were diagnosed with occupied lesions. CSF analysis indicated that the patients had low CSF glucose and high protein levels postoperatively. The mortality of culture-positive PCNSIs was 8%, which was approximately 16 times greater than the mortality of inpatients in general (0.49%, 32/6500).

**Table 1:** Demographic and clinical characteristics of 62 patients with post-neurosurgical bacterial meningitis

Characteristic	Value
Demographic parameters	
Mean age (yr)	42.5
Sex, male:female	33:29
Interval between infection and the initial neurosurgery (median day)	9 (IQR^a^ = 9)
Days in hospital (median)	46.5 (IQR = 38)
Death	5 (8%)
Operation types	
Endonasal trans-sphenoidal approach	28 (45.2%)
Craniotomy	21 (33.9%)
Shunt placement	9 (14.5%)
Decompressive craniectomy	4 (6.4%)
CSF data	
Leukocyte count (10^6/L)	media*n* = 599.5 (IQR = 3573)
Glucose level (mmol/L)	median = 2.5 (IQR = 1.8)
Lactate level (mmol/L)	media*n* = 119.5 (IQR = 7.5)
Protein level (g/L)	media*n* = 1.3 (IQR = 2.8)

^a^*IQR* interquartile range

As Table 2 shows, the distributions of Gram-positive, Gram-negative and Eumycetes were 56, 40 and 4%, respectively. The predominant Gram-positive isolate was coagulase-negative staphylococci (33%), followed by *Enterococcus faecalis* (8.9%) and *Staphylococcus aureus* (7.8%). *Acinetobacter spp.* was the predominant Gram-negative organism and was isolated in 16 (17.8%) isolates, followed by *Pseudomonas aeruginosa* in 6 (6.7%) and *Klebsiella pneumonia* in 5 (5.6%).

**Table 2:** Bacteria isolated from cerebrospinal fluid (*N* = 90)

Organism	*n* (%)
Gram-positive bacteria	51 (56)
Coagulase-negative staphylococci^a^	30 (33)
*Enterococcus faecalis*	8 (8.9)
*Staphylococcus aureus*	7 (7.8)
Others^c^	6 (6.7)
Gram-negative bacteria	36 (40)
*Acinetobacter spp.*^b^	16 (17.8)
*Pseudomonas aeruginosa*	6 (6.7)
*Klebsiella pneumoniae*	5 (5.6)
*Enterobacter aerogenes*	3 (4)
*Enterobacter cloacae*	2 (2.2)
Others^d^	4 (4.4)
Eumycetes	3 (4)
*Candida albicans*	2 (2.2)
*Cryptococcus neoformans*	1 (1.1)

^a^*Staphylococcus epidermidis* (*n* = 9), *Staphylococcus haemolyticus* (*n* = 5), *Staphylococcus capitis* (*n* = 4), *Staphylococcus warneri* (*n* = 3), *Staphylococcus hominis* (*n* = 3), *Staphylococcus sciuri* (*n* = 1), *Staphylococcus saprophyticus* (*n* = 1) and other unidentified coagulase-negative staphylococci (*n* = 4)

^b^*Acinetobacter baumannii* (*n* = 14). *Acinetobacter lwoffii* (*n* = 1), *Acinetobacter pittii* (*n* = 1)

^c^Gram-positive organisms: *Aerococcus* (*n* = 2), *Corynebacterium striatum* (*n* = 2), *Streptococcus pneumonia* (*n* = 2)

^d^Gram-negative organisms: *Brevundimonas diminuta* (*n* = 1), *Escherichia coli* (*n* = 1), *Bordetella Moreno-Lopez* (*n* = 1), *Serratia marcescens* (*n* = 1)

Table 3 shows the in vitro antibiotic sensitivities of the Gram-positive isolates; 24 coagulase-negative staphylococci isolates were sensitive to linezolid (95%), teicoplanin (100%), vancomycin (100%) and quinupristin-dalfopristin. The seven *Enterococcus faecalis* isolates were all sensitive to chloramphenicol, cefoxitin, linezolid, nitrofurantoin and tigecycline, and the seven *Staphylococcus aureus* isolates were all sensitive to amoxicillin-clavulanate, linezolid, rifampicin, rifampicin, teicoplanin and vancomycin. Of the Gram-negative isolates, there were only five samples of the *Acinetobacter spp*. available for analysis, and they displayed low sensitivity to tigecycline (40%) and minocycline (33%), as Table 4 showed.

**Table 3:** Gram-positive antibiotic sensitivity rates

Organism	AMC	AMP	CLI	CHL	CIP	ERY	FOX	GEN	LNZ	LVX	NIT	PEN	RIF	SXT	TCY	TEC	TGC	VAN	OXA	QDA
Coagulase-negative staphylococci [24]	33	–	33	–	46	23	40	61	95	57	–	15	86	52	85	100	–	100	25	100
*Enterococcus faecalis* [7]	–	67	–	100	75	0	100	67	100	–	100	67	–	–	20	83	100	83	–	–
*Staphylococcus aureus* [7]	100	–	33	–	83	28	83	43	100	–	–	0	100	50	83	100		100	83	100

*AMC* amoxicillin-clavulanate, *AMP* ampiclox, *CLI* clindamycin, *CHL* chloramphenicol, *CIP* ciprofloxacin, *ERY* erythromycin, *FOX* cefoxitin, *GEN* gentamycin, *LNZ* linezolid, *LVX* levofloxacin, *NIT* nitrofurantoin, *PEN* penicillin, *RIF* rifampicin, *SXT* sulfamethoxazole-trimethoprim, *TCY* tetracycline, *TEC* teicoplanin, *TGC* tigecycline, *VAN* vancomycin, *OXA* oxacillin, *QDA* quinupristin-dalfopristin

**Table 4:** Gram-negative antibiotic sensitivity rates

Organism	AMK	CAZ	CIP	CRO	CSL	CTX	FEP	GEN	IPM	LVX	MEM	MNO	PIP	SAM	SXT	TCC	TGC	TZP
*Acinetobacter spp.*^*b*^ [13]	8	0	0	0	17	0	0	0	18	0	20	33	0	0	0	0	40	0

*AMK* amikacin, *CAZ* ceftazidime, *CIP* ciprofloxacin, *CRO* ceftriaxone, *CSL* cefoperazone-sulbactam, *CTX* cefotaxime, *FEP* cefepime, *GEN* gentamycin, *IPM* imipenem, *LVX* levofloxacin, *MEM* meropenem, *MNO* minocycline, *PIP* piperacillin, *SAM* ampicillin-sulbactam, *SXT* sulfamethoxazole-trimethoprim, *TCC* piperacillin-clavulanate, *TGC* tigecycline, *TZP* piperacillin-tazobactam

## Discussion

PCNSIs are rare but severe complications with heavy disease burden following cranial operations. They are associated with increased costs, psychological and emotional trauma and a delay in postoperative adjuvant therapies [9, 12, 18]. The literature contains differing reports of the incidence of PCNSIs. All the PCNSI patients in this study were diagnosed by CSF culture, and the incidence was approximately 0.9% (62/6500), which was similar to some previous studies [3, 19, 20] ranging from 0.3 to 4.8%. However, other studies reported higher rates of PCNSIs, ranging from 6.5 to 7.4% [9, 12, 21]. One possible explanation may be the different inclusion criteria. In this research, only culture-positive postoperation patients were included, implying a lower false-positive rate. Different types of operations may be another reason for the discrepancy between studies. About half of the patients in this study underwent surgery using the endonasal trans-sphenoidal approach, which tends to have a low incidence of infection of approximately 1–10% [22].

The culture-positive rate of infection in previous studies, approximately 50% [9, 12, 21], which was also higher than the present study (7.5%). Possible reasons for the lower positive test rate may be the use of cefuroxime as a prophylactic antibiotic and the use of therapeutic antibiotics as soon as the infection was diagnosed [14]. Another cause could be that the same species of microorganisms cultured from different isolates within 7 days were counted as a single isolate.

The mortality rate of PCNSIs dropped drastically in past years, from 34% in 2005 [23] to 19% in 2011 [5], and a recent study reported that the mortality was 1.8% [9]. Compared to the research of Shi [9], the present study showed a higher mortality of PCNSIs (8%). A possible explanation for the higher fatality rate might be that the diagnosis of PCNSIs in this study depended on the isolation of a pathogen rather than clinical signs, which may mean that many infected patients without positive cultures were excluded. Underestimating the overall number of PCNSIs would lead to overestimating the mortality. Above all, the incidence, positive-culture rate and mortality of PCNSIs vary between studies and are hard to compare due to different inclusion criteria.

The predominant organism was Gram-positive bacteria, accounting for approximately 56% of the total isolates. Coagulase-negative staphylococci was the major organism, which included *Staphylococcus epidermidis*, *Staphylococcus haemolyticus*, and *Staphylococcus capitis*. *Acinetobacter baumannii* was the most frequent causative Gram-negative agent, accounting for 44% of the total isolates. The microbiological findings in this study were consistent with the majority of previous results, with the most common organisms grown in the culture being Gram-positive [10, 23, 24]. However, in most studies, the dominant Gram-positive organism is *Staphylococcus aureus* [23, 24]. Additionally, *Acinetobacter spp.* were the most commonly isolated Gram-negative bacteria in our study [11, 12, 25, 26], whereas Enterobacteriaceae were the most common in other studies [10, 20, 23, 24].

Of the coagulase-negative staphylococci, 18 (75%) were methicillin-resistant coagulase-negative staphylococci (MRCoNS), and all were susceptible to vancomycin, teicoplanin and quinupristin-dalfopristin. The results agreed with the results of previous studies [27, 28]. Approximately 25% (2 isolates) of *Staphylococcus aureus* isolates were methicillin-resistant *Staphylococcus aureus* (MRSA), and these isolates were susceptible to vancomycin, linezolid, rifampicin and amoxicillin-clavulanate. The incidence of MRSA in our study was higher than the 15.7% incidence Sipahi, O. R. reported [29] and lower than the 40% incidence reported by Rolston, K. V [18]. M.RSA was not a significant problem in our study, as only two samples were identified. However, it has been a major concern in recent years [30]. The results of both MRCoNS and MRSA in our study indicated that the three most effective antibiotics against the MRCoNS were vancomycin, teicoplanin, and linezolid. Vancomycin was the last resort and the drug of choice to treat infections caused by Gram-positive bacteria in CNS infection. Fortunately, all the MRCoNS and MRSA were sensitive to it. However, there were two vancomycin-resistant *Enterococcus faecalis* isolates, therefore, the emergence of resistance to vancomycin could be a serious concern for PCNSIs.

Of the Gram-negative isolates, only the susceptibility of *Acinetobacter baumannii* was analyzed; the others failed due to lack of isolates. There were 13 patients with 13 *Acinetobacter baumannii* isolates, and all 13 isolates were multidrug-resistant (MDR) bacteria with resistance to 12 out of 18 antimicrobials tested. Few studies have reported data on *Acinetobacter baumannii* isolates from CSF, but the SENTRY Antimicrobial Surveillance Program reported that the frequency of *Acinetobacter* spp. was approximately 7% [31]. However, the MDR rate was amazingly high, ranging from 50 to 70%, and the rate of carbapenem-resistant *Acinetobacter baumannii* (CRAB) has risen in recent years [32]. The emergence of MDR *Acinetobacter baumannii*, known as one of the ESKAPE pathogens [33], has become a serious medical problem worldwide [34, 35]. The data presented here could provide further evidence of the widespread distribution of multidrug-resistant *A. baumannii*. This increasing incidence is of great concern considering the lack of treatment options for dealing with MDR *Acinetobacter baumannii* infections. Similar to previous study [34], tigecycline and minocycline were the most effective antibiotic for *Acinetobacter baumannii*, however the sensitivity rates in our study were only 40 and 33%, respectively. More studies should investigate the treatment strategy in the future.

Early diagnosis and the timely use of antibiotics are essential for the treatment of PCNSIs [14]. Few studies from the Chinese mainland about the causative pathogens and their drug sensitivities were available. The present study not only showed the distribution of pathogens from a region, but reflected a similar trend to that observed worldwide, which could help clinicians choose the appropriate empirical antibiotic therapy for PCNSIs. There were a number of limitations to this study. First, the data did not yield highly specific information regarding clinical variables, such as the manifestation of CNS infection and the operative part of encephalic region, and we were not able to provide insight with regards to these topics. Second, although the number of isolates in the present study was larger than some of previous [10, 11, 24, 34], there was still selection bias, and data from a single institution may not be representative of the entire population of China. Finally, the resistance feature of each isolate was not investigated at the molecular level.

## Conclusion

Postoperative central nervous system infections (PCNSIs) are a serious complication and could lead to a higher mortality rate. MRCoNS was the predominant organism isolated and was totally susceptible to vancomycin, linezolid, rifampicin and amoxicillin-clavulanate. The management of *Acinetobacter baumannii* remains a major clinical challenge with few effective antimicrobials in PCNSIs.

## Additional file

Additional file 1: Case report form. The character of Postoperative Central Nervous System Infections (PCNSIS). (DOCX 23 kb)

## Abbreviations

CLSI: Clinical and Laboratory Standards Institute
CSF: Cerebrospinal fluid
ESBL: Extended-spectrum β-lactamases
IQR: Interquartile range
MDR: Multidrug-resistant
MRCoNS: Methicillin-resistant coagulase-negative staphylococci
PCNSIs: Patients with postoperative central nervous system infections
PUMCH: Peking Union Medical College Hospital
