# Supplementary material for: Prevalence and antibiotic resistance of bacteria isolated from the cerebrospinal fluid of neurosurgical patients at Peking Union Medical College Hospital
Source: Antimicrob Resist Infect Control. 2018 Mar 20;7:41. doi: 10.1186/s13756-018-0323-3 (PMC5859784; doi:10.1186/s13756-018-0323-3)
Supplement: Supplementary file 1 — Case report form. The character of Postoperative Central Nervous System Infections (PCNSIS). (DOCX 23 kb) [file 13756_2018_323_MOESM1_ESM.docx]

CASE REPORT FORM

THE character OF postoperative central nervous system infections (PCNSIs)

| Participant Initials | \|  \|  \|  \| \| --- \| --- \| --- \| |
| --- | --- | --- | --- | --- |
| Medical Record Number | \|  \|  \|  \|  \|  \| \| --- \| --- \| --- \| --- \| --- \| |
| Participant Study Number | \|  \|  \|  \|  \|  \| \| --- \| --- \| --- \| --- \| --- \| |
| Investigator Name |  |
| Signature Date: |  |

| Participant Information | | |
| --- | --- | --- |
| Date of Birth | \| **D** \| **D** \| **M** \| **M** \| **M** \| **Y** \| **Y** \| **Y** \| **Y** \| \| --- \| --- \| --- \| --- \| --- \| --- \| --- \| --- \| --- \| | |
| Gender | _1_ Male  _2_ Female | |
| Height: _________cm | Weight:_________kg | |
| Admission Date | \| **D** \| **D** \| **M** \| **M** \| **M** \| **Y** \| **Y** \| **Y** \| **Y** \| \| --- \| --- \| --- \| --- \| --- \| --- \| --- \| --- \| --- \| | |
| Principal Diagnosis | **______________________________________________** | |
| Secondary Diagnosis | **1_______,2_______,3_______,4_______,5_______,6_______,;** | |
| Operation Name | **_______________________,_______________________** | |
| Operation Date | \| **D** \| **D** \| **M** \| **M** \| **M** \| **Y** \| **Y** \| **Y** \| **Y** \| \| --- \| --- \| --- \| --- \| --- \| --- \| --- \| --- \| --- \| \| **D** \| **D** \| **M** \| **M** \| **M** \| **Y** \| **Y** \| **Y** \| **Y** \|   **1**  **2** | |
| Operation Information | **1.Duration _________min; Blood Loss _________ml**  **2.Duration _________min; Blood Loss _________ml** | |
| ICU | _1_ Y  _2_ N | |
| Date of Fever (>38.5℃) | \| **D** \| **D** \| **M** \| **M** \| **M** \| **Y** \| **Y** \| **Y** \| **Y** \| \| --- \| --- \| --- \| --- \| --- \| --- \| --- \| --- \| --- \| | |
| Prognosis | _1_ Cure  _2_ Death | _3_ Withdrawing |
| Date of Discharge | \| **D** \| **D** \| **M** \| **M** \| **M** \| **Y** \| **Y** \| **Y** \| **Y** \| \| --- \| --- \| --- \| --- \| --- \| --- \| --- \| --- \| --- \| | |
| Phone Number |  | |

| **Clinical Laboratory Information** | | | | | | | | | | | | | | |
| --- | --- | --- | --- | --- | --- | --- | --- | --- | --- | --- | --- | --- | --- | --- |
| No | Date of report(DD/MM/YY) | CSF | | | | | | | Blood | | | | Pathogen | MT(℃) |
|  |  | Cell (10^6/L) | Leukocyte (10^6/L) | Monocyte(%) | Multicore(%) | Glucose (mmol/L) | Lactate (mmol/L) | Protein(g/L) | Leukocyte (10^9/L) | neutrophil(10^9/L) | CRP | PCT |  |  |
| 1 |  |  |  |  |  |  |  |  |  |  |  |  |  |  |
| 2 |  |  |  |  |  |  |  |  |  |  |  |  |  |  |
| 3 |  |  |  |  |  |  |  |  |  |  |  |  |  |  |
| 4 |  |  |  |  |  |  |  |  |  |  |  |  |  |  |
| 5 |  |  |  |  |  |  |  |  |  |  |  |  |  |  |
| 6 |  |  |  |  |  |  |  |  |  |  |  |  |  |  |
| 7 |  |  |  |  |  |  |  |  |  |  |  |  |  |  |
| 8 |  |  |  |  |  |  |  |  |  |  |  |  |  |  |

Page

| **Susceptibility Test Information** |
| --- |
| Paste Report |

No

| **Therapy** **Information** | | | | | | |
| --- | --- | --- | --- | --- | --- | --- |
| Date of Start(DD/MM/YY) | Date of End(DD/MM/YY) | The chemical name of Antibiotic | frequency | administration route | Based on Susceptibility Test or not | Shunt or not |
|  |  |  |  |  |  |  |
|  |  |  |  |  |  |  |
|  |  |  |  |  |  |  |
|  |  |  |  |  |  |  |
|  |  |  |  |  |  |  |
|  |  |  |  |  |  |  |
|  |  |  |  |  |  |  |
|  |  |  |  |  |  |  |
|  |  |  |  |  |  |  |
|  |  |  |  |  |  |  |
|  |  |  |  |  |  |  |
|  |  |  |  |  |  |  |

Page
